# Magnetic hysteresis and strong ferromagnetic coupling of sulfur-bridged Dy ions in clusterfullerene Dy_2_S@C_82_ †

**DOI:** 10.1039/D0QI00771D

**Published:** 2020-07-29

**Authors:** Denis Krylov, Georgios Velkos, Chia-Hsiang Chen, Bernd Büchner, Aram Kostanyan, Thomas Greber, Stanislav M. Avdoshenko, Alexey A. Popov

**Affiliations:** a Leibniz Institute for Solid State and Materials Research, Helmholtzstraße 20, 01069 Dresden, Germany; b Center for Quantum Nanoscience, Institute for Basic Science (IBS), Seoul, Republic of Korea; c Department of Medicinal and Applied Chemistry, Kaohsiung Medical University, Kaohsiung 807, Taiwan; d Physik-Institut der Universität Zürich, Winterthurerstr. 190, CH-8057 Zürich, Switzerland

## Abstract

Two isomers of metallofullerene Dy_2_S@C_82_ with sulfur-bridged Dy ions exhibit broad magnetic hysteresis with sharp steps at sub-Kelvin temperature. Analysis of the level crossing events for different orientations of a magnetic field showed that even in powder samples, the hysteresis steps caused by quantum tunneling of magnetization can provide precise information on the strength of intramolecular Dy⋯Dy inter-actions. A comparison of different methods to determine the energy difference between ferromagnetic and antiferromagnetic states showed that sub-Kelvin hysteresis gives the most robust and reliable values. The ground state in Dy_2_S@C_82_ has ferromagnetic coupling of Dy magnetic moments, whereas the state with antiferromagnetic coupling in *C*_s_ and *C*_3v_ cage isomers is 10.7 and 5.1 cm^−1^ higher, respectively. The value for the *C*_s_ isomer is among the highest found in metallofullerenes and is considerably larger than that reported in non-fullerene dinuclear molecular magnets. Magnetization relaxation times measured in zero magnetic field at sub-Kelvin temperatures tend to level off near 900 and 3200 s in *C*_s_ and *C*_3v_ isomers. These times correspond to the quantum tunneling relaxation mechanism, in which the whole magnetic moment of the Dy_2_S@C_82_ molecule flips at once as a single entity.

## Introduction

Tremendous progress in lanthanide single-molecule magnets (SMMs) during the last decade had been largely fuelled by the design of new molecules with ever-increasing magnetic anisotropy.^1^ For single-ion SMMs, ligand-field (LF) splitting has been the main parameter on which experimental and computational studies have focused until recently,^2^ although the gradual understanding of the paramount role of molecular vibrations now shifts the focus to spin–phonon interactions.^2*j*,3^ In polynuclear SMMs, exchange and dipolar interactions between lanthanide ions create a more complex structure of magnetic states than in single-ion magnets, and the presence of such coupled states introduces a strong variation in static and dynamic magnetic properties in comparison with their single-ion counterparts. The most obvious difference is the quenching of zero-field quantum tunneling of magnetization in dinuclear SMMs, which is caused by exchange biasing.^4^ On the other hand, new relaxation pathways involving low-energy exchange-excited states can appear in dinuclear SMMs and limit their SMM performance.

Aside from compounds with lanthanide-radical coupling, which can be very strong,^5^ magnetic Ln⋯Ln interactions are usually rather weak. The energy difference between the lowest states with ferromagnetically and antiferromagnetically coupled moments, Δ*E*_AFM–FM_, is a very important parameter for dinuclear SMMs, but its precise determination is not very straightforward and often relies on the fitting of magnetic data with effective spin Hamiltonians involving some parameterized forms of Ln⋯Ln interactions. EPR studies can in principle provide more precise information on the Ln⋯Ln interactions,^6^ but for lanthanide ions with strong magnetic anisotropy, such studies encounter serious difficulties and are still rare. At the same time, the orbital mechanisms behind the exchange interactions between lanthanide ions featuring strong spin–orbit coupling are rather complicated.^7^ This limits the applicability of computational modelling at the same extent and reliability as it is used now for prediction of single-ion magnetic anisotropy in lanthanide molecular magnets.

Endohedral metallofullerenes (EMFs) encaging di-lanthanide clusters bridged *via* non-metal ions X^*q*−^, such as N^3−^, S^2−^, C_2_^2−^, or O^2−^, known as clusterfullerenes,^8^ offer simple models for the studies of Ln⋯Ln interactions. Short Ln–X bonds lead to the strong magnetic anisotropy of Ln ions and the robust SMM behaviour in many Dy-clusterfullerenes.^2*k*,4*d*,9^ The ligand-field (LF) splitting is usually so large that there is no mixing of LF and exchange states, thus simplifying the analysis and allowing to focus only on the exchange excitation in the ground state LF manifold. Note that quite a different situation is found in dimetallofullerenes featuring single-electron lanthanide–lanthanide bonds and hence giant exchange interactions,^5*c*,10^ as well as in SMMs with radical bridges,^5*a*,*b*^ but we will not consider such molecules in this work.

The first dinuclear EMF-SMM Dy_2_ScN@C_80_-*I*_h_ revealed the strong influence of Dy⋯Dy interactions on the magnetic hysteresis shape in comparison with mononuclear DySc_2_N@C_80_, and indicated a considerable Δ*E*_AFM–FM_ energy of *ca*. 6 cm^−1^.^4*d*^ Since then, we studied a number of di-nuclear EMFs and found a strong variation of the strength of Dy⋯Dy interactions in them. Δ*E*_AFM–FM_ in some of those studies was determined by fitting magnetization data.^4*d*,9*h*,*k*^ In many cases it was also established that the relaxation of magnetization occurred *via* the exchange-excited state, showing Arrhenius behaviour with the barrier equal to Δ*E*_AFM–FM_.^2*k*,4*d*,9*b*,*c*,*h*,*i*^ Both approaches have certain limitations. The shape of magnetic susceptibility and isothermal magnetization curves is very sensitive to Ln⋯Ln interactions at low temperatures, but below the blocking temperature of magnetization, SMMs do not exhibit magnetic field and temperature dependence expected in the thermodynamic regime and thus cannot be used for a fitting, whereas higher-temperature curves are less sensitive to the Ln⋯Ln interaction parameters. Arrhenius barriers may be affected by the presence of concurrent relaxation mechanisms and also depend on the accuracy of the measured relaxation times. Note that determination of magnetization relaxation time *τ*_M_ is not straight-forward when *τ*_M_ is longer than 10^4^ s or falls into the gap between 0.1–1 s (the upper limit for AC magnetometry) and ∼50 s (the lower accuracy limit for DC magnetometry).

Sub-Kelvin magnetization studies can be very useful for the determination of Ln⋯Ln interaction strength even when hysteresis sets in. Freezing thermal relaxation processes leaves QTM as the main relaxation mechanism. QTM takes places only at the level crossing and thus can give direct information on the interactions and avoids the need for fitting procedures. Such measurements were performed usually with ordered single crystals,^4*a*,11^ but their utility for powder samples is not obvious since the distribution of orientations also leads to a distribution of level crossing positions. However, the recent sub-Kelvin magnetometry study of Tb_2_ScN@C_80_ (ref. 12) showed that the QTM-related features in magnetic hysteresis of a powder sample can be fairly sharp and may help in the careful description of the low-energy magnetic states in such dinuclear SMMs. Besides, such studies give access to magnetization relaxation dynamics, which would not be accessible otherwise. As mentioned, dinuclear EMF-SMMs often have thermally activated relaxation *via* the exchange-excited state down to 2 K. But how will the system behave if the temperature is low enough to freeze this process out? What is the time scale of the QTM process in which the coupled moment of two lanthanide ions flips as a single entity? In this work we apply sub-Kelvin magnetometry measurements to two isomers of sulfide clusterfullerene Dy_2_S@C_82_ to get a deeper insight into Dy⋯Dy interactions and its influence on the magnetic hysteresis and the relaxation of magnetization in these prototype dinuclear SMMs.

## Results and discussion

### Molecular structure and alignment of magnetic moments

**Fig. 1:**
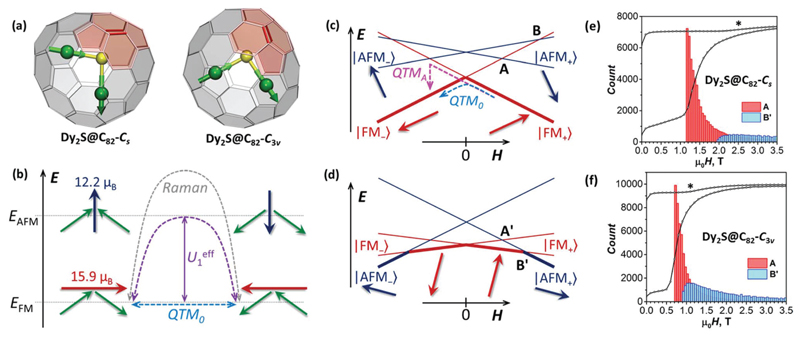
(a) Molecular structures of Dy_2_S@C_82_-*C*_s_ and Dy_2_S@C_82_-*C*_3v_ (Dy is green, S is yellow, the carbon cage is transparent gray, and green arrows show one of the possible orientations of magnetic moments of Dy ions in the ground state doublet); two fullerene isomers have different orientation of pyracelene units highlighted in light red; C–C bonds which undergo 90° rotation in Stone–Wales transformation connecting these two isomers are shown in red. (b) Schematic description of two quasi-doublets defined as ferromagnetically (FM) and antiferromagnetically (AFM) coupled, green arrows denote magnetic moments of individual Dy ions, whereas red and dark blue arrows are total moment of the Dy_2_S@C_82_ molecule, the values are computed for the Dy–S–Dy angle of 105°; dashed arrows show the main low-temperature mechanisms of the relaxation of magnetization, including quantum tunneling of magnetization (QTM), Orbach mechanism *via* AFM-coupled state with effective barrier *U*eff, and Raman mechanism *via* virtual state of higher energy. (c) and (d) show Zeeman diagrams for Dy_2_S@C_82_ for two arbitrary orientations of the molecule *versus* the magnetic field, in (c) the total magnetic moment of the FM state is close to the parallel orientation, whereas in (d) orientation is close to perpendicular; red and blue arrows show orientations of the magnetic moments for FM and AFM states, thick lines highlight the ground state in a given field range, and letters A, B, A’, and B’ mark different kinds of level crossing discussed in the text (each of these crossings is actually an avoided crossing with a certain tunneling gap, but showing this would overwhelm the figures with details). (e and f) Histograms (binning 0.05 T) of the crossing events of types A and B’ in *C*_s_ and *C*_3v_ isomers of Dy_2_S@C_82_ computed for an ensemble of 10^5^ randomly oriented molecules overlaid with experimental hysteresis curves recorded at 0.41 K.

Synthesis and structural characterization of two Dy_2_S@C_82_ isomers by single-crystal X-ray diffraction were described previously.^9*c*^ Crystallographic studies revealed *C*_s_(6) and *C*_3v_(8) isomeric structures of the fullerene cage in two EMFs. These isomers share similar cage topology and are different only in the orientation of two pyracelene units highlighted in Fig. 1a. Pseudo-rotation of one CC bond in each pyracelene unit by 90° (known as Stone–Wales transformation) interconverts the fullerene cages.

Crystallographic studies gave the Dy–S bond lengths and Dy–S–Dy angles of 2.465(5), 2.518(5) Å and 98.3(2)° in the *C*_s_ isomer and 2.437(11), 2.511(9) Å and 94.4(2)° in the *C*_3v_ isomer. But significant disorder of the metal positions may affect these values. In the molecular structures optimized at the PBE-D level with PAW 4f-in-core potentials (VASP 5.0 code^13^) the Dy–S bond lengths and Dy–S–Dy angles are 2.484, 2.509 Å and 99.1° in the *C*_s_ isomer and 2.489, 2.506 Å and 97.4° in the *C*_3v_ isomer.

Strong uniaxial ligand field imposed by sulfide ion S^2−^ leads to the orientation of Dy magnetic moments along Dy–S bonds. Different mutual orientations of Dy moments in the dinuclear cluster Dy_2_S give four states grouped into two quasi-doublets with a perpendicular orientation of the magnetic moment (Fig. 1b). The total magnetic moment of the molecule in each quasi-doublet depends on the Dy–S–Dy angle: *μ*_FM_ = 2 *μ*_Dy_ cos(*α*/2), *μ*_AFM_ = 2 *μ*_Dy_·sin(*α*/2), where *μ*_Dy_ is the magnetic moment of Dy^3+^ in the ground state, equal to 10*μ*_B_, and *α* is the angle between quantization axes of Dy ions and is approximately equal to *α* ≈ 180° − ∠(Dy–S–Dy). The equality is not rigorous here because quantization axes of Dy ions may deviate slightly from the Dy–S bond directions. Thus, for the Dy–S–Dy angle of 105°, the *μ*_FM_ and *μ*_AFM_ moments are 12.2 and 15.9*μ*_B_, respectively. In the following, the states with smaller and larger magnetic moments will be defined as anti-ferromagnetically (AFM) and ferromagnetically (FM) coupled. Note that this notation is rather arbitrary and for a Dy–S–Dy angle of 90° both moments would be equal. The preliminary study showed that the FM state in Dy_2_S@C_82_ is lower in energy than AFM.^9*c*^

### Magnetic hysteresis of Dy_2_S@C_82_ isomers

**Fig. 2:**
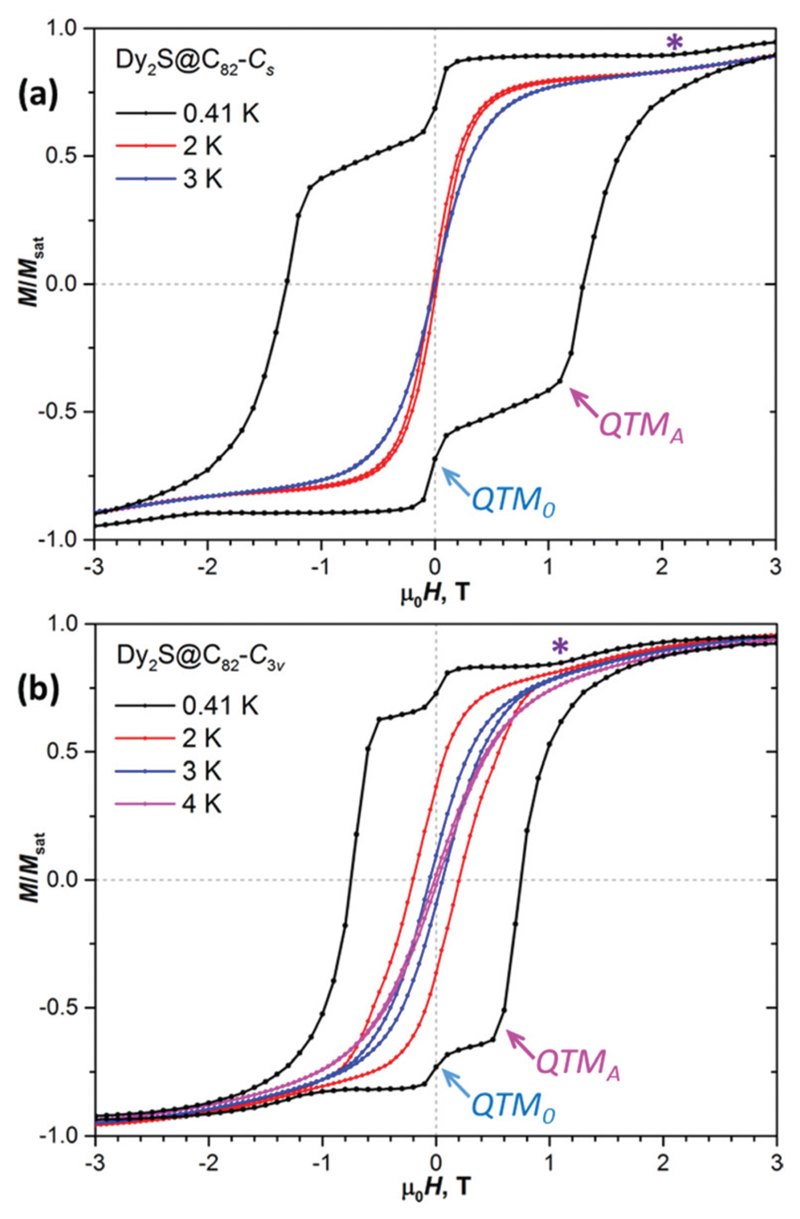
Magnetic hysteresis of (a) Dy_2_S@C_82_-*C*_s_ and (b) Dy_2_S@C_82_-*C*_3v_ measured at *T* = 0.41 K and compared to some higher-temperature curves recorded until the hysteresis is closed. Sweep rates 2.9 mT s^−1^ for *T* = 2 K and above, and 3.3 mT s^−1^ for *T* = 0.41 K. QTM_0_, QTM_A_, and asterisk denote the features appearing because of the level crossing in Zeeman diagrams and are explained in the text.

Magnetic hysteresis curves of Dy_2_S@C_82_-*C*_s_ and Dy_2_S@C_82_-*C*_3v_ are shown in Fig. 2.‡ The curves recorded at 0.41 K exhibit broad hysteresis with distinct features near zero field as well as at ±1.1 T (*C*_s_ isomer) and ±0.5 T (*C*_3v_ isomer). Measurements at 2 K give much narrower hysteresis, whereas the sharp features cannot be distinguished any more. For the *C*_s_ isomer, the hysteresis is closed completely between 2 and 3 K, while for the *C*_3v_ isomer a narrow opening is seen up to 4 K and disappears at 5 K.

The sharp features in low-*T* hysteresis curves can be associated with quantum tunnelling of magnetization (QTM). At low *T*, thermally activated relaxation processes become very slow, which makes relaxation of magnetization *via* QTM much more pronounced. As the QTM occurs at the avoided level crossing, it is necessary to understand the structure of the Zeeman diagram and possible types of level crossing events. Furthermore, the angular dependence of the Zeeman diagram needs to be understood because experimental studies are performed for powder samples with random orientation of molecules *versus* the external magnetic field. But first, it is necessary to determine the energy difference between the FM and AFM states.

### Dy⋯Dy interactions in Dy_2_S@C_82_

To determine parameters of Dy⋯Dy coupling, magnetization curves of Dy_2_S@C_82_ isomers were fitted with the effective spin Hamiltonian in eqn (1):

(1) $${\hat{H}}_{\text{spin }}={\hat{H}}_{{\text{LF}}_{1}}+{\hat{H}}_{{\text{LF}}_{2}}-2j_{12}{\hat{J}}_{1}\cdot {\hat{J}}_{2}+{\hat{H}}_{\text{ZEE}}$$

where *Ĥ*_LF_*i*__ are single-ion ligand-field Hamiltonians of Dy^3+^ with *ab initio* computed parameters, *j*_12_ is the isotropic coupling constant between dysprosium moments, and *Ĥ*_ZEE_ is the Zeeman term. Dy^3+^ moments *Ĵ*_*i*_ are treated in the |*J*,*m*_*J*_〉 basis sets of the ^6^ *H*_15/2_ multiplet. CASSCF(9,7)/SO-RASSI calculations reported earlier^9*c*^ showed that the single-ion ground states of Dy^3+^ ions in both Dy_2_S@C_82_ isomers are almost pure states with *m*_*J*_ = ±15/2. The second Kramers doublet (KD) states are calculated to be 220–290 cm^−1^ higher in energy than the ground state. Thus, the low-temperature magnetization behaviour of Dy_2_S@C_82_ molecules is predominantly determined by the single *J*_*z*_ state of Dy^3+^ ions and their coupling giving a manifold of four states described in Fig. 1b, for which Hamiltonian in eqn (1) gives a reasonable description. The energy difference between FM and AFM-coupled states following from eqn (1) is Δ*E*_AFM–FM_ = 225*j*_12_ cos(*α*), where *α* is the angle between quantization axes of two Dy^3+^ ions as introduced above.

**Fig. 3:**
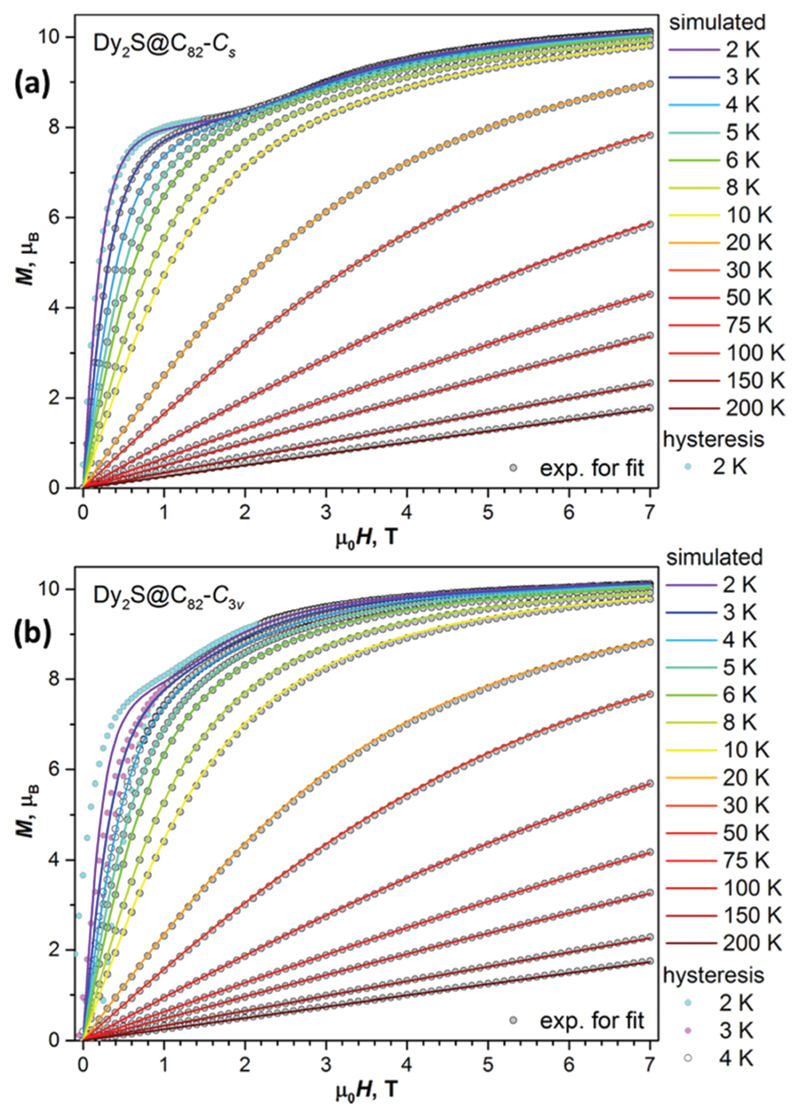
Equilibrium magnetization curves of (a) Dy_2_S@C_82_-*C*_s_ and (b) Dy_2_S@C_82_-*C*_3v_ measured at temperatures between 2 K and 200 K. Grey dots are experimental values used in the fitting procedure; coloured lines are simulated for powder samples using fitted *j*_12_ and *α* parameters (*j*_12_ = 0.16 cm^−1^ and *α* = 72.3° for Dy_2_S@C_82_-*C*_s_; *j*_12_ = 0.12 cm^−1^, *α* = 75.7° for Dy_2_S@C_82_-*C*_3v_). Coloured dots are the fragments of experimental magnetization curves with open hysteresis; these points were not used in the fitting procedure and are shown here to guide the eye. Note that the absolute experimental values of magnetization are not known because of the small sample mass, and the fitting is done for the normalized magnetization curves.

The coupling constant *j*_12_ and angle *α* are determined by fitting the experimental magnetization curves to eqn (1) taking powder-averaging into account using the PHI code^14^ (Fig. 3). In both molecules, the FM coupling is found in the ground state. For Dy_2_S@C_82_-*C*_s_, the best fit is obtained for *j*_12_ = 0.160 *±* cm^−1^ and *α* = 72.3 *±* 0.1°, which gives Δ*E*_AFM–FM_ = 11.0 cm^−1^. For Dy_2_S@C_82_-*C*_3v_, the optimal *j*_12_ is 0.117 *±* cm^−1^ and *α* = 75.7 *±* 0.3°, amounting to Δ*E*_AFM–FM_ = 6.4 cm^−1^. Assuming that Dy^3+^ moments are aligned exactly along the Dy–S bonds, these fits give ∠(Dy–S–Dy) angles of 107.7° in *C*_s_ and 104.3° in *C*_3v_ isomers, which is somewhat higher than single-crystal values and DFT predictions for the lowest energy cluster positions. However, in real structures with disordered positions of Dy_2_S clusters inside fullerenes, the ∠(Dy–S–Dy) angles are not single-valued, and the fits give only an average.

### Level crossing, QTM, and shape of magnetic hysteresis in Dy_2_S@C_82_

With determined Δ*E*_AFM–FM_ values and angles between quantization axes of Dy^3+^ ions, Zeeman diagrams can be modelled at different angles between the external field and Dy_2_S@C_82_ molecules to better understand the features observed in hysteresis curves. The analysis below follows the approach applied recently in the study of the low-temperature hysteresis in Tb_2_ScN@C_80_.^12^

If magnetic field is aligned parallel to the magnetic moment of the FM (or AFM) state, only this state is split by the field, whereas the AFM (or FM) is not affected. The situation for an arbitrary orientation of the field is shown in Fig. 1c and d. In Fig. 1c, orientation of the total magnetic moment of the FM state is close to but not exactly parallel to the field direction. One of the FM doublet components is the ground state of the molecule in the whole field range (|FM_+_〉 for *H* > 0 and |FM_−_〉 for *H* < 0). The crossing at zero field causes the QTM within the FM doublet, |FM_+_〉 ↔ |FM_−_〉 (denoted as QTM_0_ in Fig. 1b, c and 2). In this process, magnetic moments of both Dy ions flip at once. This is a low-probability process and can be observed only at very low temperature, when faster thermal processes are frozen out. Indeed, a sharp but not strong drop of magnetization can be seen upon zero field crossing in 0.41 K hysteresis curves of both Dy_2_S@C_82_ isomers, but this feature is not seen at 2 K and above (Fig. 2).

Another level crossing event of high importance for the understanding of the hysteresis shape is denoted as type A in Fig. 1c. It corresponds to the crossing of the higher-energy FM state with a lower-energy AFM state (*e.g*., the crossing of |FM_+_〉 with |AFM_−_〉 in the negative field, or |FM_−_〉 with |AFM_+_〉 in the positive field). Consider evolution of the system in Fig. 1c when the magnetic field is swept from large positive to large negative values. At *H* > 0, |FM_+_〉 is the ground state and magnetization is slowly decreasing because of the partial population of other states. At low temperatures, when the thermal relaxation is very slow, magnetization remains almost constant until zero field. During zero-field crossing, the |FM_+_〉 state can relax to the |FM_−_〉 state by the QTM_0_ mechanism. Fig. 2 shows that only ∼15–20% of Dy_2_S@C_82_ molecules undergoes the QTM_0_ and adopts the |FM_−_〉 state after crossing zero field, whereas the large part remains in the |FM_+_〉 state, resulting in the positive magnetization in the negative field. For this large part of Dy_2_S@C_82_ molecules, the fast relaxation of magnetization is triggered at the next level crossing of type A between |FM_+_〉 and |AFM_−_〉 states (also denoted as QTM_A_ in Fig. 1c and 2). The |FM_+_〉 → |AFM_−_〉 transition appears to be much more efficient than |FM_+_〉 → |FM_−_〉 as evidenced by the abrupt drop of magnetization to negative values beyond the level crossing. It is not clear yet if the QTM_A_ event results in a concerted relaxation to the |FM_−_〉 state,^4*b*,12^ or that the |AFM_−_〉 state is accessed first and then gradually relaxes to the |FM_−_〉 state. But the second option would require a thermally activated mechanism, and hence the concerted mechanism is more probable. If after crossing of type A, a part of the molecules still stays in the |FM_+_〉 state, the next crossing would be of type B with the |AFM_+_〉 state. It may also trigger the change of the magnetization *via* |FM_+_〉 → |AFM_+_〉 transition, but we do not see corresponding features in hysteresis curves (Fig. 2). Presumably, relaxation of magnetization at the level crossing of type A is very efficient, and the fraction of Dy_2_S@C_82_ molecules surviving in the |FM_+_〉 state beyond this level crossing is very small.

Another possibility not considered yet in our analysis is that the magnetic field is oriented nearly perpendicular to the magnetic moment of the FM state. In this case, |AFM_+_〉 and |AFM_−_〉 may become ground states at some large positive and negative fields, respectively (Fig. 1d). Upon reducing the field, |FM_+,−_〉 states become lower in energy than |AFM_+,−_〉 giving the level crossing of type B′ (Fig. 1d). Again, a stepwise drop of magnetization is possible at this crossing following the |AFM_+_〉 ↔ |FM_+_〉 and |FM_−_〉 ↔ |AFM_−_〉 transitions, but it cannot be as pronounced as for type A because the fraction of molecules undergoing this type of crossing in the available field range of [−7, 7] T is relatively small as discussed below. The change of magnetization at the level crossing of type B′ should also occur in the thermodynamic regime, when the relaxation of magnetization is fast. Corresponding features can be identified in magnetization curves recorded below 3–4 K (Fig. 3).

Powder samples such as studied in this work have molecules in different orientations. Therefore, the level crossing event of each type will not occur in one particular field but will be distributed over a certain field range. Depending on the shape of this distribution, QTM features in hysteresis curves may appear as sharp or smeared. To analyse the distributions, we used *j*_12_ and *α* parameters determined from the fits to experimental magnetization curves and calculated level crossing positions for Dy_2_S@C_82_ molecules with 10^5^ different orientations of the magnetic field vector around them uniformly distributed on the Fibonacci sphere.§ Fig. 1e and f show histograms of level crossing events of types A and B′ for *C*_s_ and *C*_3v_ isomers of Dy_2_S@C_82_ in the field range of 0–3.5 T.

For Dy_2_S@C_82_-*C*_s_, 58.3% molecules have the crossing of type A between 0 and 7 T, and 42% of these crossings happen between 1.16 and 1.36 T. Likewise, 57.5% of Dy_2_S@C_82_-*C*_3v_ molecules undergo this type of crossing between 0 and 7 T, and 54% of those events fall into the narrow field range between 0.72 and 0.92 T. Thus, crossing events of type A have a very sharp distribution with the asymmetric peak near the smallest field, at which this event can take place. This threshold field (*H*_A_) is simply proportional to the energy difference between AFM and FM states:

(2) $$\mu_{0}H_{\text{A}}\left[T\right]=\frac{\text{Δ}E_{\text{AFM}-\text{FM}}}{2\mu_{\text{Dy}}}=1.07\frac{\text{Δ}E_{\text{AFM}-\text{FM}}\left[{\text{cm}}^{-1}\right]}{\mu_{\text{Dy}}\left[\mu_{\text{B}}\right]},$$

where *μ*_0_ *H*_A_ is in Tesla, Δ*E*_AFM–FM_ in cm^−1^, and *μ*_Dy_ = 10*μ*_B_ is magnetic moment of a Dy^3+^ ion in Dy_2_S@C_82_, and the numerical coefficient appears because of the unit conversion. The very high density of crossing events near this threshold field translates into sharp QTM_A_ features in the magnetic hysteresis curves (Fig. 1 and 2), which allows accurate estimation of Δ*E*_AFM–FM_. Importantly, the determination of Δ*E*_AFM–FM_ from the QTM_A_ field for powder samples does not involve the angle between magnetic moments of Dy^3+^ ions *α* and is not affected by the powder averaging. Note that unlike the crossing of type B′ discussed below, the crossing of type A is an intrinsic SMM feature and can be observed in magnetization curves only when relaxation of magnetization near zero field is slow enough to enable a significant non-equilibrium fraction of molecules in the state |FM_+_〉 in the negative field or in the state |FM_−_〉 in the positive field.

The crossing of type B′, on the other hand, has a less distinct position (Fig. 1e and f). For Dy_2_S@C_82_-*C*_s_, 25% of all molecules have this kind of crossing in the field range below 7 T. The smallest field, in which the crossing can take place, is 1.95 T, and the next 0.5 T (1 T) range includes only 16% (33%) of events among the molecules with the B′ point below 7 T. For Dy_2_S@C_82_-*C*_3v_, the distribution is slightly denser. The fraction of all molecules with B′ crossing below 7 T is 32%, of that 22% (43%) have this crossing in the range of 0.5 T (1 T) above the threshold field of 1.14 T. Thus, in contrast to type A, the crossing of type B′ occurs with a smaller fraction of molecules (hence smaller change of magnetization), and the distribution of the events in the field scale is much broader. Although the corresponding deflection can be seen in the experimental curves (marked with an asterisk in Fig. 1e, f and 2), we cannot determine if this feature occurs because of the QTM-induced relaxation at the level crossing, or because the system simply follows a thermodynamic regime. The lowest field, at which B′ crossing can take place, is defined in eqn (3):

(3) $$\mu_{0}H_{{\text{B}}^{\prime }}\left[T\right]\;=\frac{\text{Δ}E_{\text{AFM}-\text{FM}}}{2\mu_{\text{Dy}}\sin\left(\alpha /2\right)}=1.07\frac{\text{Δ}E_{\text{AFM}-\text{FM}}\left[{\text{cm}}^{-1}\right]}{\mu_{\text{Dy}}\left[\mu_{\text{B}}\right]\sin\left(\alpha /2\right)}=\frac{\mu_{0}H_{\text{A}}}{\sin(\alpha /2)},$$

Eqn (3) also allows determination of Δ*E*_AFM–FM_ if *α* is known or can be estimated from the known ∠(Dy–S–Dy) angle. But because of the broad distribution of crossing events in powder samples, and because the maximum in the distribution is shifted from the threshold field *H*_B′_ (Fig. 1e and f), the corresponding features in magnetization curves are very smeared, and precision of the Δ*E*_AFM–FM_ value estimated this way for powder samples would be not very high. Anyway, analogs of formulae (3) were used earlier for estimation of exchange interaction in powder samples, mainly for dinuclear complexes with the AFM ground state.^4*e*,15^ For oriented single crystals though, the value can be quite accurate.^4*a*,11^

### Magnetization relaxation times

**Fig. 4:**
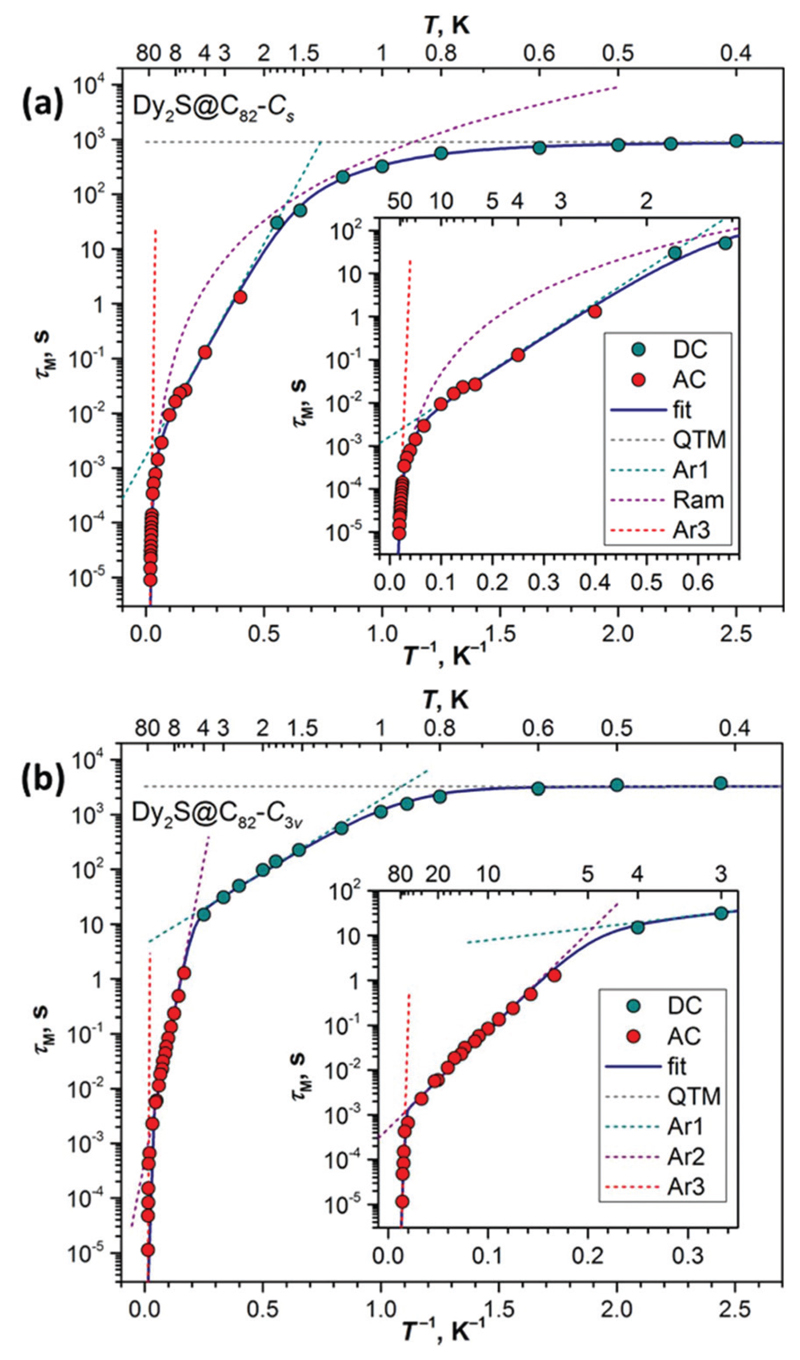
Magnetization relaxation times of (a) Dy_2_S@C_82_-*C*_s_ and (b) Dy_2_S@C_82_-*C*_3v_. Dark cyan and red dots are DC and AC measurements, solid lines are fits by a combined equation eqn (1), and dashed lines are contributions of QTM, Raman, and Arrhenius processes. The insets show magnification of higher-temperature parts.

Low-temperature magnetic studies allowed the determination of magnetization relaxation times and a more complete description of magnetization dynamics in Dy_2_S@C_82_ isomers. Fig. 4 shows the temperature dependence of zero-field relaxation times (*τ*_M_) in the whole available temperature range, including the data determined by recording decay curves with DC magnetometry in this work and relaxation times determined by AC magnetometry earlier in ref. 9*c*. Both isomers exhibit several distinct relaxation regimes, which are described by a combined equation:

(4) $$\tau_{\text{M}}^{-1}\left(T\right)=\tau_{\text{QTM}}^{-1}+CT^{n}+\sum_{i}\tau_{0i}^{-1}\exp\left(-U_{i}^{\text{eff}}/T\right),$$

where *τ*_QTM_ is the QTM relaxation time, the second term describes the relaxation of magnetization *via* the Raman mechanism, and the third term describes one or more Arrhenius regimes. Using the DC measurements from this work and earlier AC data, we refitted the *τ*_M_-*T* ^−1^ dependencies.

At the lowest temperatures, relaxation time tends to level off, which indicates a transition to the QTM relaxation regime. Characteristic QTM times obtained from the fits are 906 ± 80 s for the *C*_s_ isomer and 3224 ± 418 s for the *C*_3v_ isomer. These rather long times explain why the QTM_0_ regime can be observed in hysteresis curves only at sub-Kelvin temperature. Consider the FM ground-state of a dinuclear Dy system such as shown in Fig. 1b. If a magnetic moment of one of the metal ions is flipped, the system arrives at the AFM state, which has higher energy. Thus, the Δ*E*_AFM–FM_ difference prevents zerofield QTM with the flipping of one Dy^3+^ moment. At the same time, it allows the thermally activated relaxation process with the barrier $U_{1}^{\text{eff }}$ equal to Δ*E*_AFM–FM_ (Fig. 1b). We observed this kind of mechanism in many di-nuclear EMFs studied before,^2*k*,4*d*,9*b*,*c*,*h*,*i*,*k*,12^ and it can be also recognized in Dy_2_S@C_82_. For the *C*_s_ isomer, this mechanism dominates between 2 and 10 K, the $U_{1}^{\text{eff }}$ barrier is 17.9 ± 0.5 K, whereas attempt time *τ*_01_ is 1.6 ± 0.2 ms. In the *C*_3v_ isomer, the barrier is lower, $U_{1}^{\text{eff }}$ = 6.1 ± 0.4 K, but attempt time is much longer, *τ*_01_ = 4 ± 1 s, and the mechanism is operative between 1 and 4 K. Thus, only the use of sub-K temperatures in this work allowed freezing out the Orbach relaxation *via* the AFM state and we observed the |FM_+_〉 ↔ |FM_−_〉 QTM regime, in which the whole magnetic moment of the Dy_2_S@C_82_ molecule flips at once (Fig. 1b).

At higher temperature, the mechanism of relaxation changes to Raman in the *C*_s_ isomer with *C* = (1.8 ± 3) 10^−3^ s^−1^ K^−*n*^, *n* = 4.0 ± 0.1. For *C*_3v_ we observe instead another Arrhenius process, with $U_{2}^{\text{eff }}$ of 50 ± 2 K and *τ*_02_ of (4.9 ± 0.7) 10^−4^ s. The Raman mechanism with a strong coupling to certain low frequency vibration modes, such as endohedral cluster vibrations, is likely to be the reason for this linear regime.^16^ These Raman mechanisms govern relaxation of magnetization of Dy_2_S@C_82_ up to 40–50 K. Above this temperature, another change of the relaxation mechanism takes place for the *C*_3v_ isomer. This time the Orbach mechanism with the relaxation *via* ligand-field excited states of Dy^3+^ ions is likely to play the main role, and we obtain the barrier as high as 1569 ± 180 K. For the *C*_s_ isomer the fitting at high temperature is more ambiguous (see Fig. S6†). Similar to the *C*_3v_ isomer, we can also invoke one more Arrhenius process, which would have a barrier of 683 ± 83 K. At the same time, the Raman process alone also gives a reasonable description of the data. But since the χ″ signal by these temperatures dropped dramatically and the values were obtained at the limit of the magnetometer sensitivity, the reliability of the determined relaxation times is unfortunately low, which also affects stability and reliability of the fit. We thus prefer to restrain from the further discussion of these barriers.

### Dy⋯Dy interactions in Dy_2_S@C_82_ and comparison to other dinuclear Dy molecular magnets

In this work we could make estimations of Δ*E*_AFM–FM_ in Dy_2_S@C_82_ by three independent methods: from the QTM_A_ features in sub-Kelvin magnetic hysteresis curves (Fig. 2), from the fit of magnetization curves (Fig. 3), and as the barrier $U_{1}^{\text{eff }}$ in the low-temperature different EMF types are caused by the strong variation of Arrhenius regime (Fig. 4). The values for the two isomers are compared in Table 1. For the *C*_s_ isomer, we observe a good agreement of all three methods, giving the numbers in the range of 10.7–12.4 cm^−1^. For the *C*_3v_ isomer, the absolute value is smaller and hence the difference between estimations of 4.2 cm^−1^ from $U_{1}^{\text{eff }}$ to 6.4 cm^−1^ from the fit of magnetization curves is comparably large. The latter seems to be an overestimation since the distribution of crossing events computed with the Δ*E*_AFM–FM_ value from the fit of *M*–*H* curves has the maximum at a somewhat higher field than the QTM_A_ feature in the hysteresis curve (Fig. 1f). Overall, estimation of the Δ*E*_AFM–FM_ value from the QTM_A_ features in sub-Kelvin hysteresis appears to be the most straightforward and reliable. A possible contribution of other relaxation mechanisms or errors in the determined relaxation times can affect the $U_{1}^{\text{eff }}$ value, whereas the fit of magnetization curves in the case of open magnetic hysteresis has to rely on higher-temperature data, which are less sensitive to the value of Δ*E*_AFM–FM_.

The Dy⋯Dy coupling energy can be further divided into exchange and dipolar contributions, $\text{Δ}E_{\text{AFM}-\text{FM}}^{\text{dip}}$ and $\text{Δ}E_{\text{AFM}-\text{FM}}^{\text{dip}}$. The dipolar term can be computed exactly when the Dy⋯Dy distance and orientation of magnetic moments are known. Using the angle from the fit of magnetization curves and Dy–S bond lengths from DFT calculations, $\text{Δ}E_{\text{AFM}-\text{FM}}^{\text{dip}}$ values are estimated as 2.2 cm^−1^ in *C*_s_ and 2.3 cm^−1^ in *C*_3v_ isomers. $\Delta E_{\text{AFM-FM }}^{\text{exch }}$, calculated as the difference between total and dipolar interaction energies, therefore is 8.5 cm^−1^ in *C*_s_ and 2.8 cm^−1^ in *C*_3v_ isomers (the total energy estimated from hysteresis is used hereafter).

Table 2 compares the values of Δ*E*_AFM–FM_, $\text{Δ}E_{\text{AFM}-\text{FM}}^{\text{dip}}$, and $\text{Δ}E_{\text{AFM-FM }}^{\text{exch }}$ from this work to those of other dinuclear Dy metallofullerenes studied earlier,^9*c*,*h*,*i*,*k*^ including Dy_2_O, Dy_2_C_2_ and Dy_2_MN (M = Sc, Lu) clusterfullerenes with bridging O^2−^, C_2_^2−^, and N^3−^ units. Nitride and carbide clusterfullerenes also exhibited FM interactions between Dy ions, Dy_2_C_2_@C_82_ showing the largest Δ*E*_AFM–FM_ value of 12.1 cm^−1^ (determined from $U_{1}^{\text{eff }}$ in ref. 9*c*). The Δ*E*_AFM–FM_ energy in Dy_2_S@C_82_-*C*_s_ is comparable to this value. Oxide clusterfullerenes with Dy_2_O clusters tend to show AFM or weak to negligible FM interactions, Dy_2_O@C_82_-*C*_2v_ featuring the largest Δ*E*_AFM–FM_ gap of −12.9 cm^−1^. Importantly, all EMF-SMMs have very similar $\text{Δ}E_{\text{AFM}-\text{FM}}^{\text{dip}}$ energies, and large variations in total Dy⋯Dy interaction energies across different EMF types are caused by the strong variation of the exchange term.

To put these values into a broader context of di-lanthanide molecular magnets, we took into account that the most frequently used approach to describe Dy⋯Dy interactions nowadays employs the pseudospin model popularized by Ungur and Chibotaru in their POLY_ANISO code.^21^ The ground magnetic state of Dy^3+^ ions is described as a pseudospin *s̃* = 1/2 with a highly anisotropic *g*-tensor (close to (0, 0, 20) for the Kramers doublet with dominant *m*_*J*_ = ±15/2 term), and the pseudospin exchange Hamiltonian within the Lines model^22^ takes the form of eqn (3):

(5) $${\hat{H}}_{\text{exch }}=-J_{\text{tot}}{\hat{\hat{s}}}_{1}\cdot {\hat{\hat{s}}}_{2}=-\left(J_{\text{dip }}+J_{\text{exch }}\right)\hat{{\hat{s}}_{1}}\cdot \hat{{\tilde{s}}_{2}}$$

With this Hamiltonian, Δ*E*_AFM–FM_ = 0.5*J*_tot_ cos(*α*), and hence *J*_tot_ = 450*j*_12_ (where *j*_12_ is the coupling constant from eqn (1)). The calculated *J*_tot_, *J*_dip_, and *J*_exch_ constants for dinuclear EMF-SMMs are listed in Table 2. The *J*_tot_ and *J*_exch_ values of Dy_2_S@C_82_-*C*_s_, 70.4 and 56.0 cm^−1^, are the largest among all EMF-SMMs.

We are aware of only two molecular magnets with sulfurbridged Dy ions other than the Dy_2_S@C_82_: Dy_4_ complex with thiolate ligand bridges,^23^ and the dinuclear complex with Dy(Cp′)_2_ units bridged *via* two (μ-SSiPh_3_) groups.^17^ In both systems, Dy⋯Dy coupling is weakly AFM as can be assumed based on the shape of χ*T* curves. The Δ*E*_AFM–FM_ and *J*_tot_ values in {Cp′_2_Dy(μ-SSiPh_3_)}_2_ are −2 and −4.4 cm^−1^, respectively.

The μ_2_-O bridges are much more common than μ_2_-S in dinuclear Dy molecular magnets, especially in the form of {Dy_2_O_2_} fragments. For those, we found only three compounds with |*J*_tot_| exceeding 10 cm^−1^ (Table 2; see ref. 9*i* for a recent Dy_2_S@C_82_ or observed earlier for isomers of Dy_2_O@C_82_.^9*i*^ survey of Dy⋯Dy interaction parameters in {Dy_2_O_2_} compounds). Two of them have phenoxide bridges with FM coupling and *J*_tot_ values of 15.0 and 11.4 cm^−1^.^18,19^ In the complex with vanilloyl bridges, the coupling is AFM and *J*_tot_ is −11 cm^−1^.^20^ Table 2 shows that in EMF-SMMs the range of coupling constants can be several times larger. This large difference in *J*_tot_ constants may appear somewhat misleading because the Δ*E*_AFM–FM_ values also depend on the angle between Dy axes. In EMF-SMMs, magnetic moments of Dy ions are usually non-collinear, so the range of the energies is not as high as for the coupling constants. But still, Δ*E*_AFM–FM_ energies in EMF-SMMs can be considerably larger than those in other {Dy_2_} molecular magnets.

One reason for this lies in the comparably strong dipolar interactions between Dy moments in EMFs caused by the relatively short Dy⋯Dy distance and suitable Dy–X–Dy angles maximizing dipolar interactions. Yet in many {Dy_2_O_2_} compounds, the distances are even shorter than those in EMFs. Thus, we conclude that the exchange interactions between Dy moments in EMFs are mainly responsible for these unprecedentedly strong Dy⋯Dy interactions in EMFs. The reasons for this strong exchange are not clear yet. Short Dy–X bonds leading to enhanced superexchange *via* the bridging atoms may be one of the reasons. But this factor cannot explain why variation of exchange coupling can be so strong in different cage isomers, such as that found in this work for *C*_s_ and *C*_3v_ isomers of Dy_2_S@C_82_ or observed earlier for isomers of Dy_2_O@C_82_.^9*i*^

**Fig. 5:**
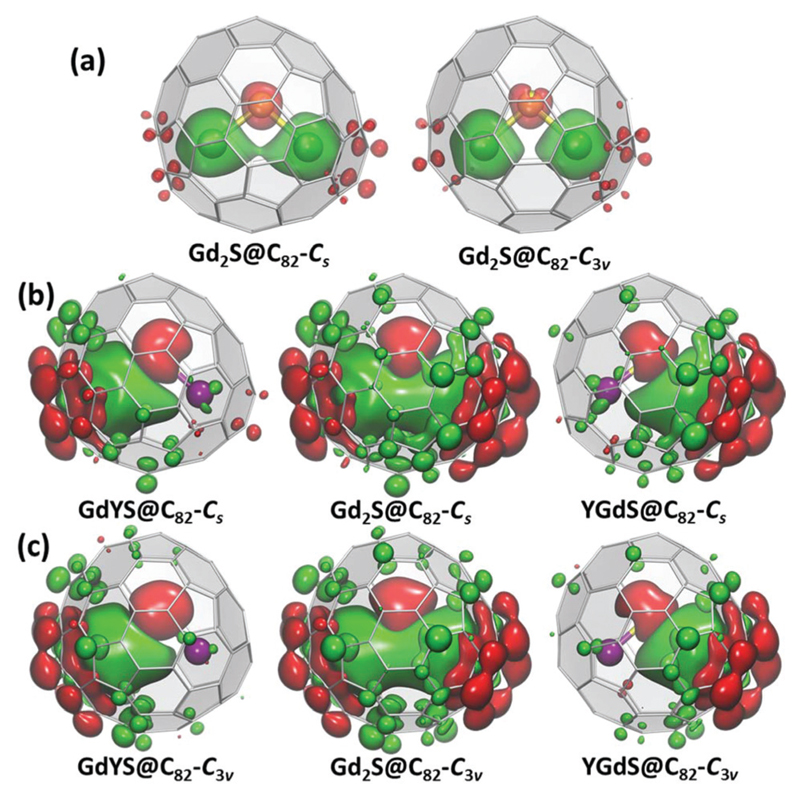
DFT-computed spin-density distribution (green – “+”, red – “−”) in: (a) Gd_2_S@C_82_-*C*_s_ and Gd_2_S@C_82_-*C*_3v_ molecules shown with isovalues of ±0.0012 a. u. (b) Gd_2_S@C_82_-*C*_s_ and GdYS@C_82_-*C*_s_ with an isovalue of ±0.00012 a. u. (c) Gd_2_S@C_82_-*C*_3v_ and GdYS@C_82_-*C*_3v_ with an isovalue of ±0.00012 a. u. The isosurfaces in (a) are plotted semitransparent to show positions of metals and sulfur in the endohedral cluster. Computations performed at the PBE0/TZVP level with DKH scalar-relativistic correction and DKH-tailored full-electron basis sets.

Evidently, the fullerene cage in EMF-SMMs should not be considered as just a container for magnetic species. We suggest that the interaction between Dy ions is also affected through the spin polarization of the fullerene π-system in the spirit of the Ruderman–Kittel–Kasuya–Yosida (RKKY) mechanism of interaction between magnetic atoms *via* conduction electrons in metals.^24^ For instance, the RKKY mechanism explains oscillatory distance dependence of interactions between magnetic adatoms in graphene, an infinite limit of the fullerene π-system.^25^ Fig. 5 plots spin density distribution in Gd_2_S@C_82_ (*S* = 15) and GdYS@C_82_ (*S* = 8) molecules computed at the PBE0 level with full-electron basis sets.¶ The use of Gd instead of Dy in these calculations allows the application of a single-determinant DFT approach and limits the focus to spin-only contribution to the Ln⋯Ln exchange interactions; spin–orbit coupling effects cannot be captured by this simple approach. Calculations for Gd_2_S@C_82_ also allow using brokensymmetry DFT to estimate the exchange coupling between Gd magnetic moments (see ref. 9*c* and ESI†), but such results cannot be directly transferred to Dy analogs.

When the isovalues of ±0.0012 a. u. are used in plotting the spin density (*ρ*_spin_) isosurfaces, the surface with positive *ρ*_spin_ (coloured green in Fig. 5a) encompasses two Gd atoms as can be expected for the state with spin multiplicity of *S* = 15. At the same time, a pronounced negative spin polarization of the bridging sulfur is also well seen. Obviously, superexchange *via* the μ_2_-S atom should be considerable in these systems. Besides, the negative spin polarization of fullerene carbon atoms closest to Gd is also visible. The plots with lower spin density isovalues of ±0.00012 a. u. show that spin polarization of carbon atoms with alternating sign extends over the whole fullerene cage. The negative spin polarization of carbons near Gd is changed to the positive one for more distant carbons. Interestingly, although the cage spin polarization patterns calculated for GdYS@C_82_ molecules resemble closely halves of spin-density plots in Gd_2_S@C_82_, they are not completely identical (Fig. 5b and c). Besides, Gd-induced spin polarization of the cage carbons in GdYS@C_82_ extends to the half of the cage not coordinated to Gd. Thus, there should be non-negligible through-cage interaction between endohedral lanthanide ions. It is reasonable to suggest that through-cage spin–spin interaction pathways should depend on the topology of the fullerene π-systems, and thus be different from cage to cage even when structural parameters of the endohedral cluster are very similar. Further exploration of this effect is worth a detailed study but goes beyond the scope of this work.

## Conclusions

In this work, we performed a study of Dy⋯Dy magnetic interactions and low-temperature relaxation dynamics in Dy_2_S@C_82_ as a prototype dinuclear SMM with a ferromagnetically coupled ground state. Although the study is performed on powder samples, the broad sub-Kelvin magnetic hysteresis with clear QTM steps observed for both cage isomers of Dy_2_S@C_82_ appeared instrumental for the determination of the energy difference between the FM and AFM states. Comparison with the values determined by other approaches, such as fitting of magnetization data or the energy barrier of the Arrhenius relaxation process, showed reasonable agreement. Comparison to other dinuclear SMMs revealed that the *C*_s_(6) isomer of Dy_2_S@C_82_ features one of the highest Dy⋯Dy coupling strength values ever reported. Furthermore, the twofold variation of the Dy⋯Dy coupling strength between two cage isomers is found. This variation cannot be explained by the difference in the structural parameters of the Dy_2_S cluster in two structures and points to a possibility of the indirect exchange interactions between lanthanide ions *via* the fullerene π-system.

Measurements of the magnetization relaxation time at subKelvin temperatures also allowed achieving a relaxation regime not observed in dinuclear EMF-SMMs before. Typically, the main low-temperature relaxation mechanism in these compounds is the Orbach process with the barrier corresponding to the energy difference between FM and AFM states. In Dy_2_S@C_82_ this mechanism is observed down to 1–2 K. But below 1 K, this thermally activated process becomes inefficient, giving way to quantum tunnelling with the simultaneous flip of two Dy moments.

## Supplementary Material

^†^Electronic supplementary information (ESI) available. See DOI: 10.1039/d0qi00771d

SI

## Figures and Tables

**Table 1:** Parameters of Dy⋯Dy interactions in Dy_2_S@C_82_ isomers determined by different methods^a^

EMF	Δ*E*_hyst_	$U_{1}^{\text{eff }}$	Δ*E*_fit_	*α*_fit_
Dy_2_S(a)C_s2_-*C*_s_	10.7 ± 0.5	12.4 ± 0.4	11.0	72.3 ± 0.1
Dy_2_S@C_S_2-*C*_3v_	5.1 ± 0.5	4.2 ± 0.3	6.4	75.7 ± 0.3

a Δ*E*_hyst_, $U_{1}^{\text{eff }}$, and Δ*E*_fit_ are estimations of Δ*E*_AFM–FM_ (in cm^−1^), respectively, from the QTM_A_ feature in sub-Kelvin hysteresis (Fig. 2), from the Arrhenius regime in relaxation times (Fig. 4), and from the fit of magnetization curves (Fig. 3). The latter also gives *α*_fit_ as the angle between magnetic moments of Dy^3+^ ions

**Table 2:** Energies and pseudospin coupling constants of Dy⋯Dy interactions in Dy_2_S@C_82_ isomers compared to those in some other dinuc-lear EMF-SMMs and {Dy_2_} compounds^a^

EMF*^b^*	Δ*E* ^tot^	Δ*E* ^dip^	Δ*E* ^exch^	*J*_tot_	*J*_dip_	*J*_exch_
Dy_2_S(a)C_82_-*C*_s_	10.7	2.2	8.5	70.4	14.4	56.0
Dy_2_S@C_82_-*C*_3v_	5.1	2.3	2.8	41.3	18.5	22.8
Dy_2_O@C_72_-*C*_s_	1.5	3.0	–1.5	4.0	8.0	–4.0
Dy_2_O@C_74_-*C*_2_	~0.1	2.6	–2.5	0.2	5.1	–4.9
Dy_2_O(a)C_82_-*C*_s_	–7.5	3.0	–10.5	–23.3	9.3	–32.6
Dy_2_O@C_82_-*C*_3v_	–5.4	2.5	–7.8	–21.6	10.2	–31.8
Dy_2_O@C_82_-*C*_2v_	–12.9	2.6	–15.6	–41.9	8.6	–50.5
Dy_2_C_2_(a)C_82_-*C*_s_	12.1	2.6	9.5	64.4	13.6	50.8
Dy_2_ScN@C_80_-*I*_h_	5.6	3.3	2.3	24.9	14.5	10.4
Dy_2_LuN@C_80_-*I*_h_	3.0	3.3	–0.3	12.6	14.0	–1.4
{Cp′_2_Dy(μ-SR)}_2_	–2.0			–4.4	–2.2	–2.2
{Dy_2_O_2_}-A	7.0			15.0	5.5	9.5
{Dy_2_O_2_}-B	6.0			11.4	4.6	6.8
{Dy_2_O_2_}-C	–5.3			–11.0	–2.7	–8.4

a Δ*E* ^tot^ is Δ*E*_AFM–FM_ (in cm^−1^), whereas Δ*E* ^dip^ and Δ*E* ^exch^ are dipolar and exchange contributions, respectively, and Δ*E* ^exch^ is computed as Δ*E* ^tot^ – Δ*E* ^dip^; *J*_tot_, *J*_dip_ and *J*_exch_ are pseudospin coupling constants (in cm^−1^) from eqn (5).

b Δ*E* ^tot^ for Dy_2_S@C_82_ is determined from hysteresis in this work, or as $U_{1}^{\text{eff }}$ for Dy_2_O@C_2n_ (ref. 9i and k), Dy_2_C_2_@C_82_ (ref. 9c), and Dy_2_MN@C_80_ (ref. 9h). In {Cp′_2_Dy(μ-SR)}_2_ from ref. 17, R = SiPh_3_; {Dy_2_O_2_}-A is [Dy_2_(dbm)_2_(LH_2_)_2_]·H_2_O from ref. 18 (LH_3_ = (1E,3E)-2-hydroxy-5-methylisophthalaldehyde dioxime, Hdbm = dibenzoyl-methane); {Dy_2_O_2_}-B is [Dy(L)Cl(CH_3_OH)]_*n*_ from ref. 19 (H_2_L = *N*′-(5-bromo-2-hydroxybenzylidene)pyrazine-*N*-oxide-carbohydrazide); {Dy_2_O_2_}-C is [Dy_2_(a’povh)_2_(OAc)_2_(DMF)_2_] from ref. 20 (H_2_a’povh = *N*′-[amino (pyrimidin-2-yl)methylene]-*o*-vanilloyl hydrazine).

## Footnotes

‡ The magnetization was measured in a Quantum Design MPMS3 vibrating sample magnetometer (VSM) with a ^3^He cryostat.

§ The ensemble of Zeeman diagrams was computed for 10^5^ different orientations of the magnetic field vectors evenly spaced along with the Fibonacci sphere grid points, assuming Hamiltonian eqn (1). The massive data were generated and analyzed using in-house Python scripts while the Hamiltonian eigenproblems were solved using the PHI code.^14^

¶ DFT calculations for Gd_2_S@C_82_ and GdYS@C_82_ molecules were performed with the Orca 4.2.1 suite^26^ using the PBE0 functional, DKH scalar-relativistic correction, and SARC-TZVP basis sets.^27^ Spin-densities are visualized with VMD.^28^
