# Supplementary material for: Magnetic hysteresis and strong ferromagnetic coupling of sulfur-bridged Dy ions in clusterfullerene Dy2S@C82 †
Source: Inorg Chem Front. Author manuscript; Available in PMC 2021 Jan 12. (PMC7116581; doi:10.1039/D0QI00771D)
Supplement: SI [file EMS109248-supplement-SI.pdf]

Electronic Supplementary Material (ESI) for Inorganic Chemistry Frontiers.
This journal is © the Partner Organisations 2020

Magnetic hysteresis and strong ferromagnetic coupling of sulfur-bridged Dy ions in clusterfullerene $Dy_2S@C_{82}$

Denis Krylov,[ab] Georgios Velkos,[a] Chia-Hsiang Chen,[ac] Bernd Büchner,[a] Aram Kostanyan,[d] Thomas Greber,[d] Stanislav M. Avdoshenko,[a] and Alexey A. Popov*[a]

Supporting Information

Experimental and calculated magnetization curves S2

Magnetization relaxation times of $Dy_2S@C_{82}$ S4

Fitting of relaxation times S6

Crystal-field splitting in $Dy_2S@C_{82}$ S10

Broken-symmetry calculations of di-Gd analogs S11

Experimental and calculated magnetization curves

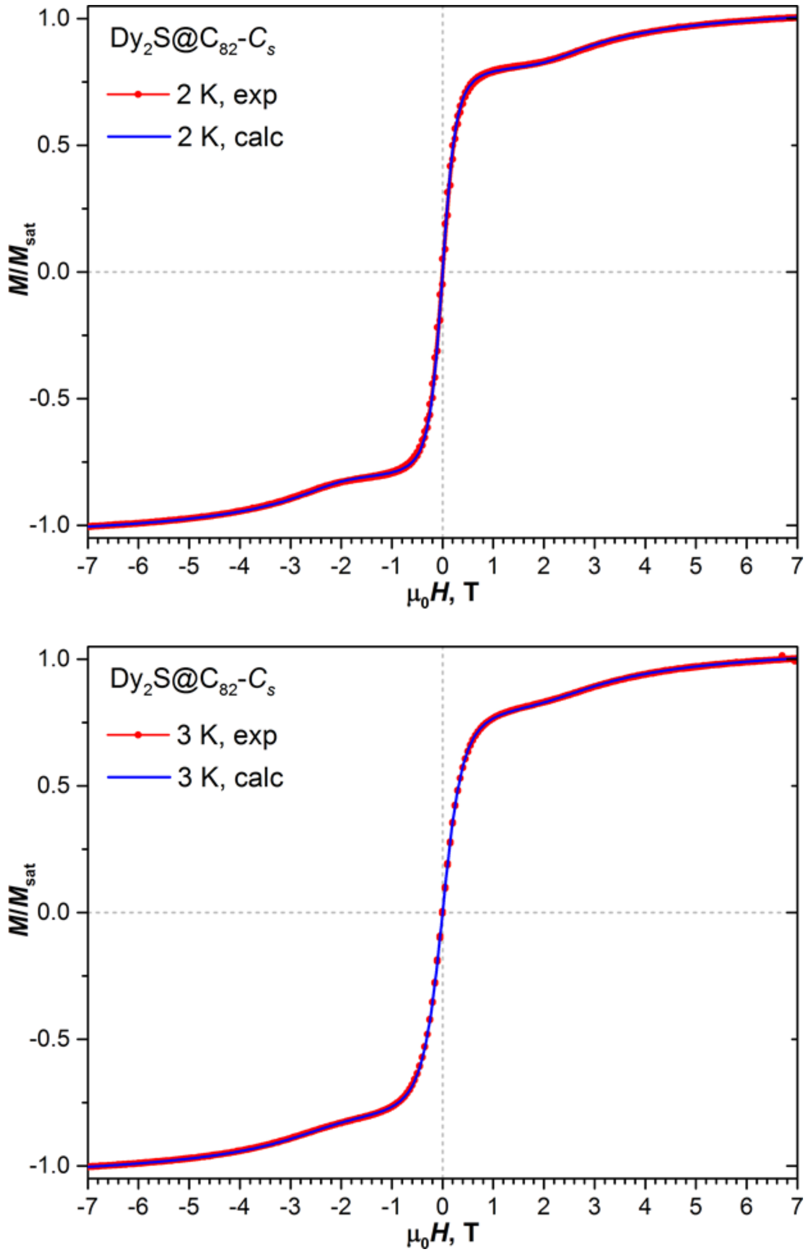

Figure S1. Experimental (red) and calculated (blue) magnetization curves of $Dy_2S@C_{82}$-C_s at 2 K and 3 K. Calculations are performed with j_{12} and α parameters obtained in the fit described in the main text.

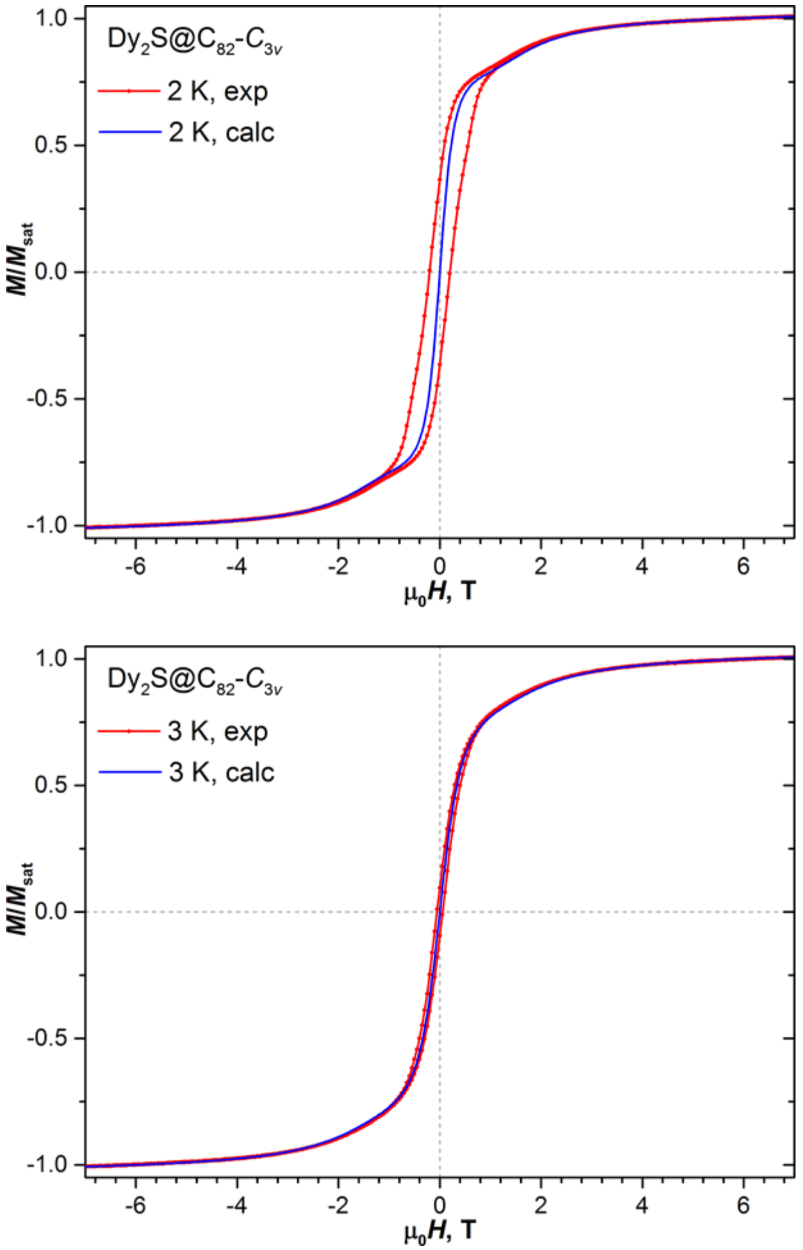

Figure S2. Experimental (red) and calculated (blue) magnetization curves of $Dy_2S@C_{82}$-C_{3v} at 2 K and 3 K. Calculations are performed with j_{12} and α parameters obtained in the fit described in the main text. Despite the hysteresis in experimental curves, it can be seen that the inflections due to the level crossing of type B' happen in experimental and calculated curves at the same positions. Of course, computed equilibrium curve and experimental curve with an open hysteresis cannot coincide.

Magnetization relaxation times of $Dy_2S@C_{82}$

Relaxation times of magnetization τ_M were determined from the magnetization decay curves recorded after the fast sweep of the magnetic field from 3 T to a zero field. Decay curves are shown in Fig. S3. Magnetization decay curves was then fitted with stretched exponential function:

$$M(t) = M_{eq} + (M_0 - M_{eq}) \exp\left[-\left(\frac{t}{\tau_M}\right)^{\beta}\right] \quad (S1)$$

Where M_{eq} and M_0 are the equilibrium and initial magnetizations, respectively, τ_M is a characteristic relaxation time and β is an additional parameter that corresponds to the time-dependent decay rate. The values of τ_M and β are listed in Tables S1a and S1b, whereas Fig. S4 and S5 show experimental and fitted curves for each temperature.

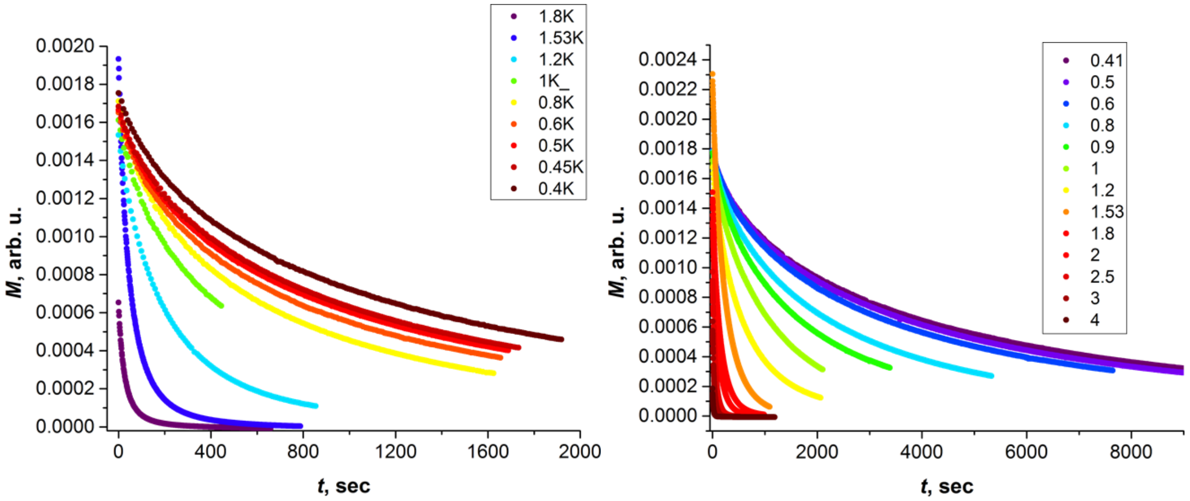

Figure S3. Magnetization decay curves of $Dy_2S@C_{82}$-C_s (left) and $Dy_2S@C_{82}$-C_{3v} (right)

Table S1a. Magnetization relaxation times and β parameters of $Dy_2S@C_{82}$-C_s

T, K	τ_M, s	±	β	±
0.40	945.0	8.8	0.695	0.004
0.45	833.5	8.4	0.702	0.004
0.50	797.2	6.1	0.702	0.003
0.60	707.5	4.8	0.703	0.003
0.80	566.6	5.1	0.678	0.005
1.00	324.6	15.2	0.826	0.015
1.20	206.7	0.5	0.837	0.003
1.53	50.4	0.2	0.739	0.003
1.80	29.9	0.3	0.715	0.005

Table S1b. Magnetization relaxation times and β parameters of $Dy_2S@C_{82}$-C_{3v}

T, K	τ_M, s	±	β	±
0.41	3686.1	6.7	0.649	0.001
0.50	3413.3	3.2	0.662	0.001
0.60	2966.3	4.9	0.672	0.001
0.80	2104.8	3.4	0.709	0.001
0.90	1573.5	4.4	0.745	0.001
1.00	1131.1	4.7	0.800	0.002
1.20	566.4	0.9	0.835	0.002
1.53	225.5	0.1	0.840	0.000
1.80	140.1	0.1	0.861	0.001
2.00	97.1	0.1	0.858	0.001
2.50	49.8	0.1	0.849	0.002
3.00	31.0	0.1	0.856	0.002
4.00	14.9	0.1	0.851	0.004

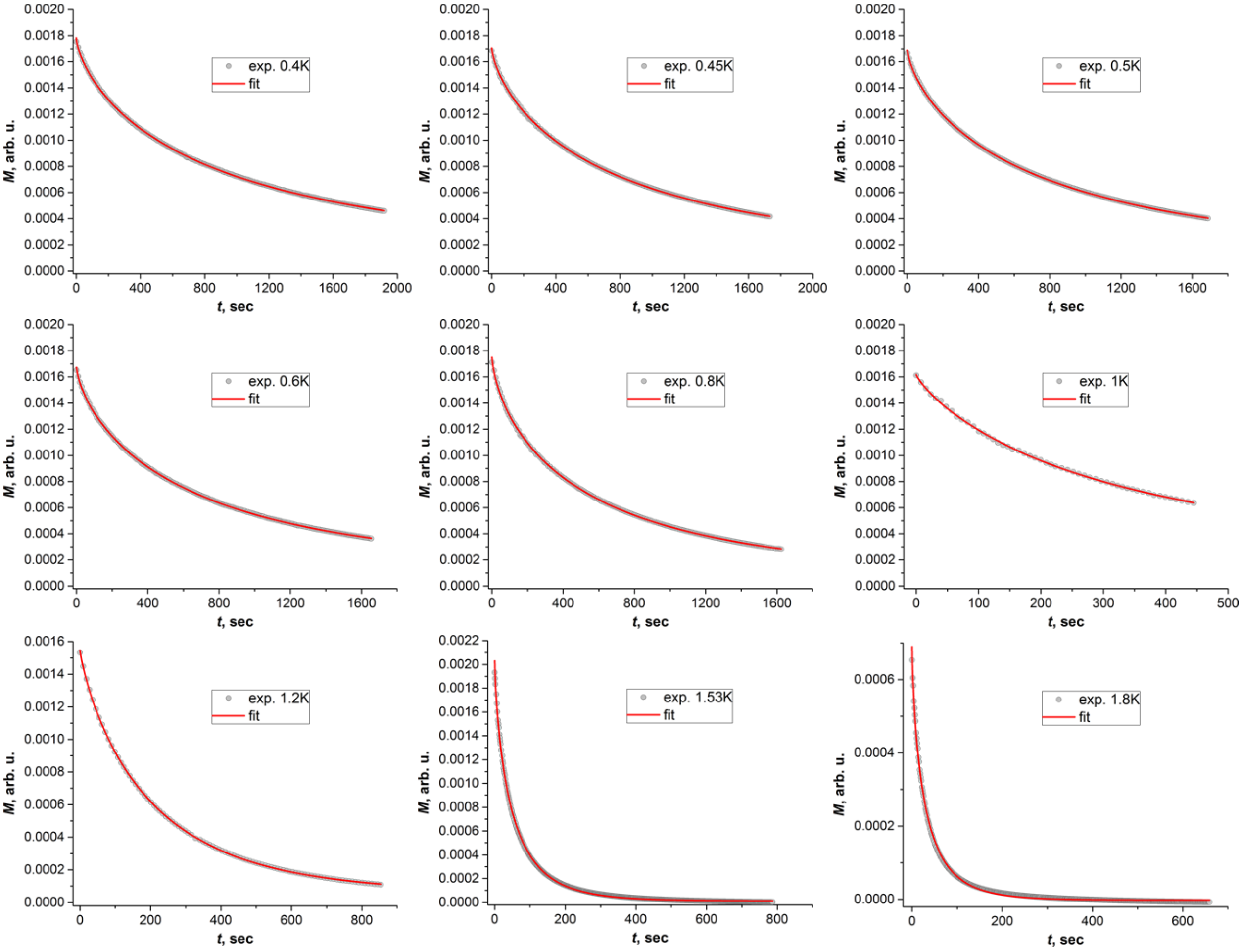

Figure S4. Decay of magnetization in $Dy_2S@C_{82}$-C_s measured at different temperatures (dots) and fits with stretched exponentials (red curves)

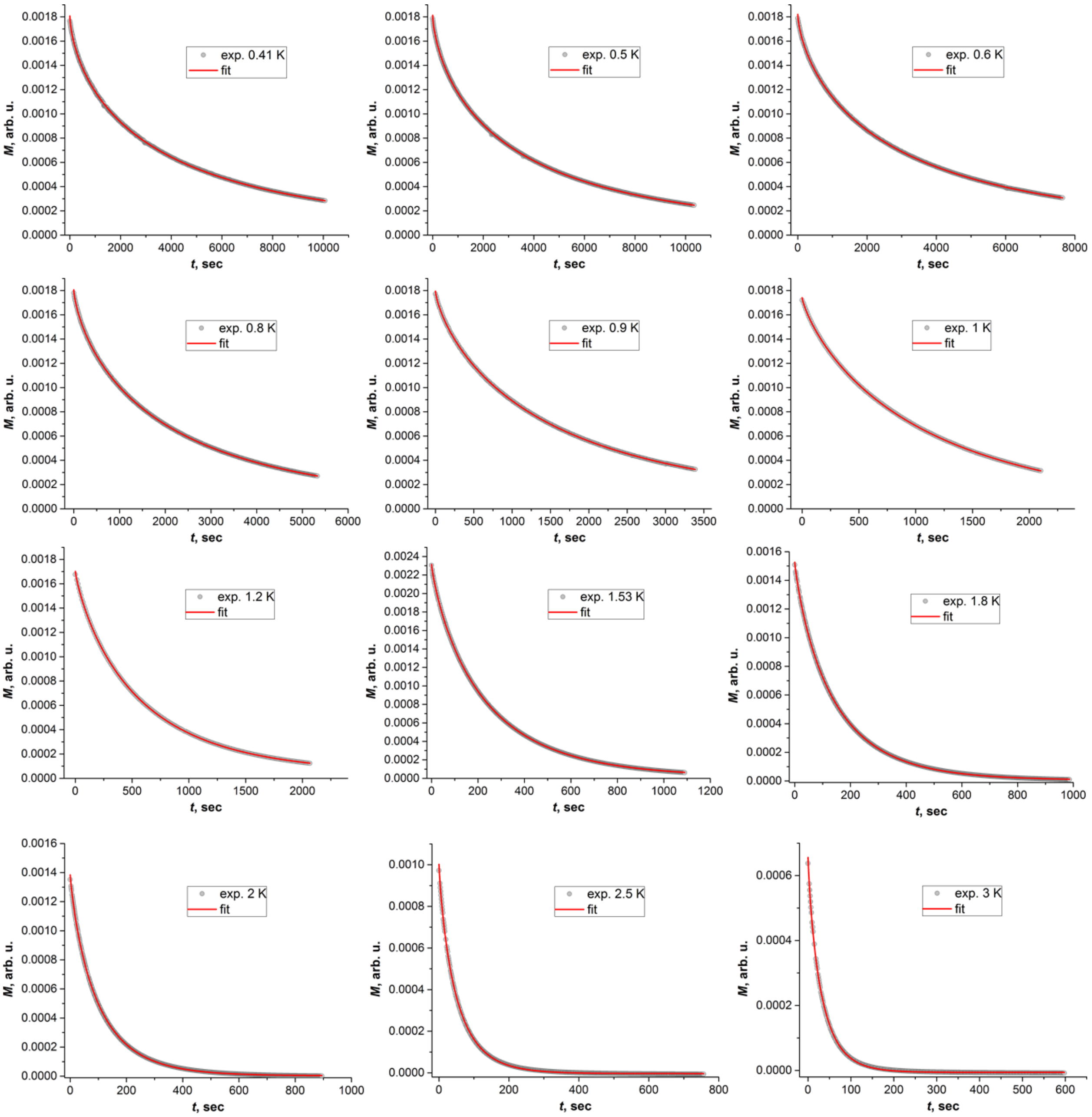

Figure S5. Decay of magnetization in $Dy_2S@C_{82}$-C_{3v} measured at different temperatures (dots) and fits with stretched exponentials (red curves)

Fitting of relaxation times

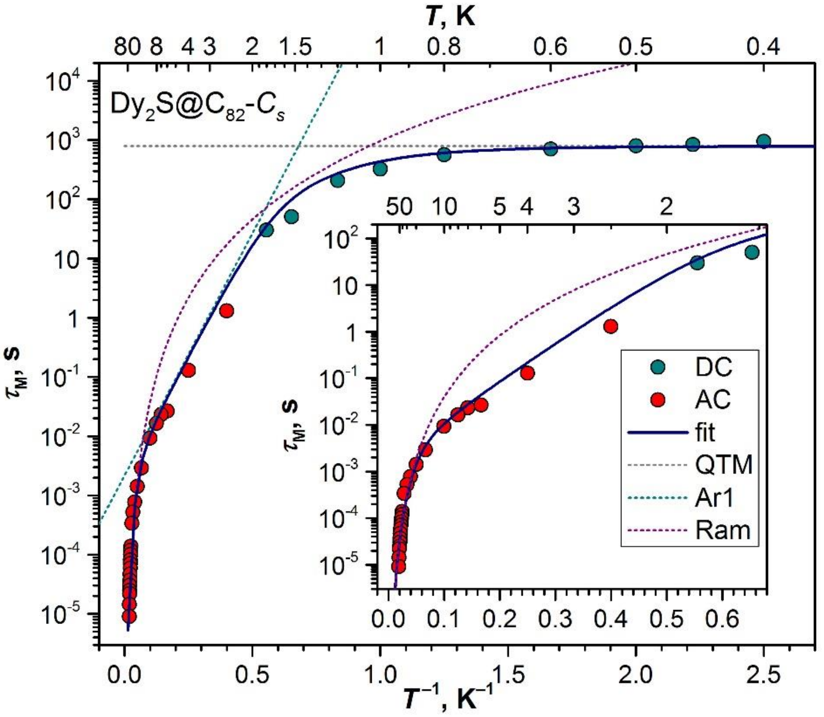

Figure S6a. Fitting of relaxation times of C_s isomer with QTM, Raman, and Arrhenius processes:

$$\tau_{\mathrm{M}}^{-1}(T) = \tau_{\mathrm{QTM}}^{-1} + CT^n + \tau_{01}^{-1}\exp(-U_1^{\mathrm{eff}}/T),$$

Fitted parameters are:

τ_{QTM} = 793±158 s,

C = (1.0±0.6) 10^{-3} $s^{-1}K^{-n}$, n = 4.39±0.14

τ_{01} = (2.2±0.6) 10^{-3} s, U_1^{eff} = 16.8±1 K

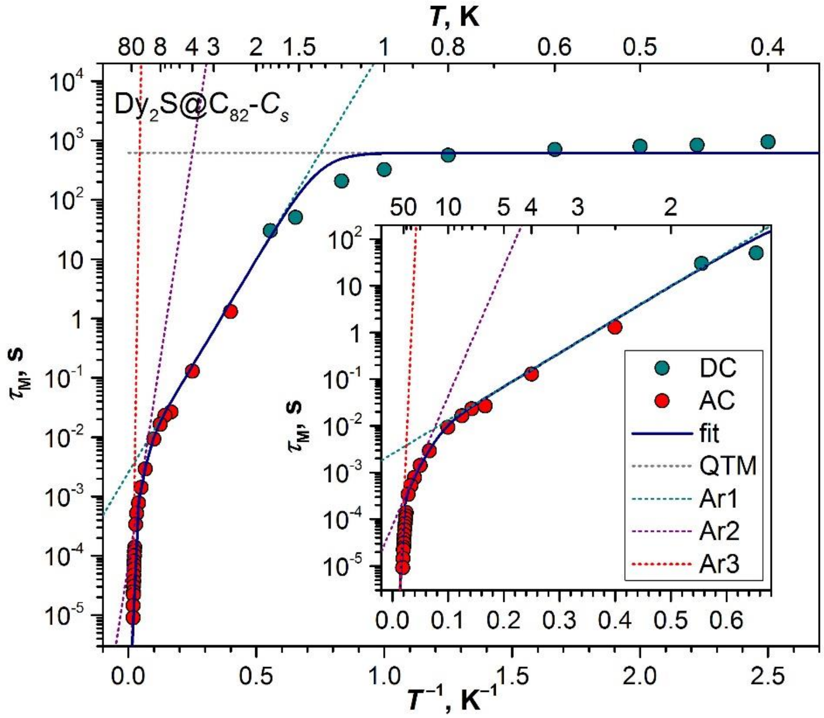

Figure S6b. Fitting of relaxation times of C_s isomer with QTM, and three Arrhenius processes:

$\tau_{\mathrm{M}}^{-1}(T) = \tau_{\mathrm{QTM}}^{-1} + \sum_i \tau_{0i}^{-1}\exp(-U_i^{\mathrm{eff}}/T)$,

Fitted parameters are:

τ_{QTM} = 618±67 s,

τ_{01} = (2.5±0.6) 10^{-3} s, U_1^{eff} = 15.7±0.6 K

τ_{02} = (7.2±4.3) 10^{-5} s, U_2^{eff} = 63.9±13.8 K

τ_{03} = (6.7±9) 10^{-10} s, U_3^{eff} = 519±67 K

Crystal-field splitting in $Dy_2S@C_{82}$ as computed ab initio at the CASSCF/RASSI level in Ref. [1]

Table S3a: $Dy_2S@C_{82}$-C_s, Dy1

state	g_x	g_y	g_z	*E, cm*$^{-1}$
1	0.0002	0.0003	19.9650	0.0
2	0.0841	0.1302	17.0652	230.7
3	0.7347	0.9324	13.5916	380.2
4	0.5223	1.7535	10.5329	507.2
5	0.9391	3.2439	8.0043	644.9
6	3.8986	5.0971	7.6777	765.1
7	1.9265	3.6483	14.6724	850.9
8	0.2946	0.5340	19.6667	912.1

Table S3b: $Dy_2S@C_{82}$-C_s, Dy2

state	g_x	g_y	g_z	*E, cm*$^{-1}$
1	0.0032	0.0038	19.7650	0.0
2	0.0035	0.0044	17.0344	221.6
3	0.0207	0.0448	14.3340	447.4
4	0.1675	0.2221	11.3647	620.5
5	2.0854	2.2280	8.1007	721.4
6	2.4208	2.5672	5.4223	797.3
7	6.7258	5.4888	1.5485	856.9
8	0.7307	4.5589	15.8178	906.7

Table S4a: $Dy_2S@C_{82}$-C_{3v}, Dy1

state	g_x	g_y	g_z	*E, cm*$^{-1}$
1	0.0003	0.0005	19.8411	0.0
2	0.0251	0.0318	17.3085	267.8
3	0.3805	0.4850	14.3019	423.1
4	1.3665	1.8706	11.3274	547.2
5	2.0251	3.8422	9.8286	650.6
6	8.1833	5.8844	0.6785	740.4
7	2.8356	4.9427	11.3273	805.0
8	0.5616	2.1737	17.4047	882.2

Table S4b: $Dy_2S@C_{82}$-C_{3v}, Dy2

state	g_x	g_y	g_z	*E, cm*$^{-1}$
1	0.0006	0.0007	19.8566	0.0
2	0.0229	0.0280	17.2624	293.4
3	0.3115	0.3856	14.1632	458.2
4	0.5479	1.0213	11.2205	590.4
5	2.5290	3.4277	9.4193	699.0
6	8.4728	5.0828	0.3987	786.0
7	2.1471	5.2201	12.3239	856.7
8	0.2711	0.8407	18.5977	967.9

Broken-symmetry calculations of di-Gd analogs

Exchange coupling parameters j_{12}^{ex}(Gd-Gd) in Gd-EMF analogs of di-Dy EMFs were computed in Ref. [1] using Orca code at the PBE0/TZVP-DKH level within the broken-symmetry approximation, they correspond to the Hamiltonian:

$$H = -2j_{12}S_1 \cdot S_2$$

Table S5. Exchange coupling parameters in Gd analogs of di-Dy EMFs.

	j_{12}^{ex}(Gd-Gd), cm^{-1}
$Gd_2S@C_{82}$-C_s	1.24
$Gd_2S@C_{82}$-C_{3v}	0.64
$Gd_2O@C_{82}$-C_{3v}	0.31

Thee data predict larger coupling constant for C_s isomer tan for C_{3v}, which agrees with the order of values we found experimentally for $Dy_2S@C_{82}$ isomers. At the same time, we would like to note that broken-symmetry DFT also predict ferromagnetic interactions of Gd ions in $Gd_2O@C_{82}$, whereas experimental data point to the antiferromagnetic coupling of Dy ions in $Dy_2O@C_{82}$.[2] Thus, it is not clear if the results for $Gd_2S@C_{82}$ can be transferred to $Dy_2S@C_{82}$.

References

1. Chen, C.-H.; Krylov, D. S.; Avdoshenko, S. M.; Liu, F.; Spree, L.; Yadav, R.; Alvertis, A.; Hozoi, L.; Nenkov, K.; Kostanyan, A.; Greber, T.; Wolter, A. U. B.; Popov, A. A., Selective arc-discharge synthesis of Dy_2S-clusterfullerenes and their isomer-dependent single molecule magnetism. *Chem. Sci.* **2017**, *8*, 6451-6465.
2. Yang, W.; Velkos, G.; Liu, F.; Sudarkova, S. M.; Wang, Y.; Zhuang, J.; Zhang, H.; Li, X.; Zhang, X.; Büchner, B.; Avdoshenko, S. M.; Popov, A. A.; Chen, N., Single Molecule Magnetism with Strong Magnetic Anisotropy and Enhanced Dy···Dy Coupling in Three Isomers of Dy-Oxide Clusterfullerene $Dy_2O@C_{82}$. *Adv. Sci.* **2019**, *6*, 1901352.
